# Beyond the Edit: Integrating CRISPR‐Cas Genome Editing Into the Cereal Breeding Pipeline

**DOI:** 10.1155/ijog/1457684

**Published:** 2026-10-01

**Authors:** Baber Ali, Aqsa Hafeez, Nijat Imin

**Affiliations:** ^1^ Faculty of Engineering Computing and Science Western Sydney University Penrith New South Wales Australia westernsydney.edu.au; ^2^ Division of Plant Sciences Research School of Biology Australian National University Acton Australian Capital Territory Australia anu.edu.au; ^3^ Hawkesbury Institute for the Environment Western Sydney University Penrith New South Wales Australia westernsydney.edu.au

**Keywords:** cereal breeding pipeline, CRISPR-Cas genome editing, doubled haploid production, freedom to operate, marker-assisted selection, multiplex editing

## Abstract

Cereal breeding must deliver complex trait combinations under a changing climate, and CRISPR‐Cas editing is presented as the technology that will accelerate this. Existing reviews document the editing toolkit thoroughly, yet few follow an edited allele through the steps separating an edited plant from a released cultivar. This review addresses that gap by treating genome editing as one module within a cereal breeding pipeline and asking, at each interface, whether integration has been demonstrated or only proposed. Evidence was selected against three criteria, namely, that the work was performed in a cereal, that the outcome was measured rather than projected and that genotype and environment were stated. Applying this standard across wheat, rice, maize and barley yields a more qualified picture than current summaries suggest. Multiplex editing is the dominant strategy for polygenic traits, yet recovery of a complete multilocus genotype is limited by the least efficient guide rather than the average efficiency. Doubled haploid production shortens fixation only where several loci segregate independently, and its induction, identification and doubling steps impose losses that offset part of the saving. Marker‐assisted selection acts on backcross progeny and on haploid inducers rather than among near‐isogenic edited events, and genomic selection combined with editing rests on livestock simulations whose assumptions cereals do not meet. Transformation efficiency, licensing of regeneration technology and jurisdiction‐specific approval remain binding constraints. Every module has been demonstrated in some cereal. The full sequence has not and closing that gap needs integration studies rather than further gains in editing precision.

## 1. Introduction

Wheat, rice, maize and barley together supply a large share of global dietary energy, and their continued productivity under a changing climate depends on breeding methods capable of introducing and fixing complex trait combinations within practical timeframes [1]. CRISPR‐Cas editing has moved from proof‐of‐concept mutagenesis toward targeted modification of genes and regulatory elements, supported by a toolkit that extends from Cas9 to Cas12 and Cas13 orthologs and to base and prime editing [2].

Several reviews published since 2023 have covered this ground, and they differ in emphasis rather than in structure. Crop‐focused reviews catalogue trait applications and editing platforms species by species [2]. Technology‐focused reviews trace the progression from nuclease editing to base and prime editing [3]. Strategy‐focused reviews treat multiplex editing as the route to polygenic traits [4, 5]. Breeding‐focused reviews address speed breeding and genomic selection largely without reference to editing [6]. Each is thorough within its own boundary. None of these follows a single edited allele through fixation, background recovery, field validation and licensing, which are the steps that separate an edited plant from a released cultivar. Table 1 sets out this comparison and identifies the gap this review addresses.

**Table 1:** Systematic comparison of recent reviews on CRISPR‐Cas editing in cereals against the pipeline modules examined in the present review. Coverage is scored as substantial, partial or absent based on whether a module is analysed with supporting evidence, mentioned without analysis or not addressed.

Review	Primary focus	Editing toolkit	Multiplex and polygenic traits	Doubled haploid fixation	MAS and genomic selection	IP and freedom to operate	Regulatory and field validation	Crops covered
Ahmar et al. [1]	Sustainable cereal production through editing	Substantial	Partial	Absent	Absent	Absent	Partial	Wheat, rice, maize and barley
Namata et al. [2]	Crop‐specific editing applications	Substantial	Partial	Absent	Absent	Absent	Partial	Maize and sorghum
Kaniganti et al. [3]	Editing platforms and pathway reprogramming	Substantial	Partial	Absent	Absent	Absent	Partial	Wheat, rice, maize and barley
Yin and Tsai [5]	Multiplex editing for polygenic traits	Partial	Substantial	Partial	Absent	Absent	Absent	Multiple crop species
Kumar et al. [4]	Multiplex editing and trait engineering	Partial	Substantial	Absent	Partial	Absent	Absent	Multiple crop species
Ćeran et al. [6]	Genomic‐assisted speed breeding	Absent	Absent	Partial	Substantial	Absent	Partial	Multiple crop species
Present review	Editing as one module of the breeding pipeline	Partial	Substantial	Substantial	Substantial	Substantial	Substantial	Wheat, rice, maize and barley

The contribution of this review is therefore a pipeline analysis rather than a further catalogue of editing tools. It is novel in three respects. First, it treats doubled haploid production, marker‐assisted selection, genomic selection and freedom‐to‐operate constraints as parts of one pipeline and asks at which specific interface each fails rather than listing them as adjacent technologies. Second, it applies a stated evidence standard, separating demonstrated integration from proposed integration, so that the framework rests on reported outcomes rather than on plausibility. Third, it tests whether combining these modules shortens the interval to a released cultivar and reports where the evidence does not support that expectation.

Evidence was selected against three criteria applied throughout. A study was treated as direct evidence only where the work was performed in a cereal species, where the reported outcome was measured rather than projected and where the genotype and growth environment were stated. Work meeting only some of these criteria is identified as such at the point of citation. Findings transferred from dicot models or from a single reference genotype are qualified explicitly and are not presented as general properties of the crop. Proposals and conceptual schemes are named as proposals and are not counted as evidence that integration has been achieved.

Two breeding technologies are directly relevant to this question. Doubled haploid systems can compress the number of generations needed to reach a fully homozygous line. This compression is not automatic. Haploid induction rates in wheat remain low and strongly dependent on donor genotype and induction method, spontaneous chromosome doubling is incomplete, regenerated haploids are sterile until doubling succeeds and haploids must be identified against a background of noninduced seed using morphological or molecular markers [7]. Each of these steps imposes a cost that offsets part of the generations saved. Section 4 quantifies these costs for the specific cereal systems in which editing and haploid induction have been combined and asks whether the combination shortens the pipeline in practice.

Marker‐assisted selection and genomic selection are distinct approaches with different requirements, and they enter an editing pipeline at different points. Marker‐assisted selection tracks a small number of loci whose position and effect are already known, which makes it directly applicable to an edited allele of defined sequence. Genomic selection instead estimates breeding values from genome‐wide markers and requires a large training population that is both genotyped and phenotyped, so it cannot be applied to a single edited line or to a small set of near‐isogenic events [8]. The distinction matters for how each is used here. Marker‐assisted selection verifies edit transmission and recovers the recurrent parent genome when an edit generated in a transformable genotype is moved into elite germplasm. Genomic selection operates one step later, on the segregating populations produced when edited lines are crossed into a breeding programme, where variation at unedited loci is again present. Section 5 examines the published evidence for each of these two roles separately rather than treating them as a single selection step.

A third constraint operates alongside both. Access to a nuclease platform, to a morphogenic regeneration factor or to a specific trait application is governed by patents and licensing terms that differ between academic and commercial use, and licence costs can exclude programs in lower‐income regions from commercialising an edited line even when the underlying science is complete [9]. Freedom to operate is therefore treated here as a pipeline stage rather than as a closing caveat, and Section 2 separates ownership of editing systems from access to transformation and regeneration technologies.

The distance between laboratory demonstration and field‐ready cultivars is not a minor procedural step. A field trial of genome‐edited, low‐asparagine wheat carrying a knockout of *TaASN2* required separate regulatory consent, multiyear field assessment and confirmation of the intended biochemical outcome under open conditions before the line could be considered for release [10]. The interval occupied by these steps exceeded the interval needed to generate the mutant. Laboratory‐validated edits therefore do not translate automatically into field performance, and this translation step warrants dedicated analysis rather than assumption.

The remainder of the review is organised around this pipeline logic, beginning with the editing toolkit and its licensing constraints, then moving through trait applications, fixation systems, selection integration and translational constraints. Emerging computational approaches to target prioritisation and guide design are considered in Section 7, where their present evidential status in cereal breeding programs is assessed rather than assumed [11]. It returns in the conclusion to the pipeline‐integration framework introduced here. Figure 1 outlines this framework schematically, showing genome editing, doubled haploid production and marker or genomic selection as linked modules feeding into field validation and variety release, with the translational bottlenecks identified in Section 6 marked at the interfaces between modules.

**Figure 1:**
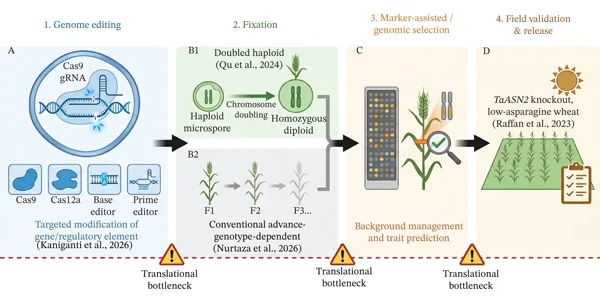
Conceptual framework depicting the integration of CRISPR‐Cas genome editing within a cereal breeding pipeline, showing four linked stages that connect an initial edited allele to a field‐validated cultivar. Stage 1, edit generation, covers nuclease, base and prime editing platforms with their delivery routes. Stage 2, allele fixation, contrasts doubled haploid production with conventional generation advance. Stage 3, background management, distinguishes marker‐assisted verification and recurrent parent recovery from population‐level genomic prediction. Stage 4 covers regulatory assessment and multiyear field validation. Return arrows mark the interfaces at which a candidate line re‐enters an earlier stage, which are examined as translational bottlenecks in Section 6.

## 2. From Editing Platform to Breeding Deployment

### 2.1. Matching the Editing Modality to the Breeding Objective

The platform decision facing a breeding programme is not which nuclease is most recent but which class of allele the target trait requires. This framing narrows the toolkit considerably. Loss of function at a susceptibility or negative‐regulator locus is achieved by a nuclease that produces small insertions or deletions through nonhomologous end joining, for which Cas9 remains the default in all four major cereals. Targets in adenine‐ and thymine‐rich sequences, or targets that must be edited simultaneously, favour Cas12a, which recognises a thymine‐rich protospacer‐adjacent motif and processes a single transcript into multiple guide RNAs without separate cassettes [12]. Reproducing a favourable natural allele that differs by one nucleotide requires base editing, and reproducing a defined small insertion or deletion requires prime editing. Requirements that are transcript‐level rather than genomic fall to Cas13, which does not produce a heritable change and therefore sits outside the breeding pipeline at present [3].

Protospacer‐adjacent motif availability determines whether the required allele is reachable at all. Engineered Cas9 variants have relaxed this constraint, generally at the cost of reduced on‐target efficiency or increased self‐editing. A chimeric variant combining the recognition domain of *Streptococcus pyogenes* Cas9 with an evolved *Streptococcus canis* Cas9 reached previously inaccessible sites in rice and produced herbicide‐resistant germplasm through adenine base editing of *OsACC* [13]. This example is relevant to breeding because it delivered a usable allele rather than a methodological demonstration and because it combined motif engineering with base editing in one construct.

Base editors fuse a catalytically impaired Cas nickase to a deaminase and convert cytosine to thymine or adenine to guanine within a defined window. In cereals, they have delivered herbicide resistance, disease resistance and grain quality traits in rice, wheat and maize, but their output is constrained by motif availability, by the width of the editing window and by bystander edits at neighbouring bases inside that window [14]. Bystander edits matter for breeding rather than only for methodology because a line carrying an unintended adjacent substitution is a different allele from the natural variant the program set out to reproduce.

Prime editing couples a nickase to a reverse transcriptase and a prime editing guide RNA. This allows base substitutions, small insertions and small deletions to be written directly at the target site without relying on homology‐directed repair, which occurs at low frequency in most plant tissues. Engineering of pegRNA stability and of the reverse transcriptase, including removal of the RNase H domain of the Moloney murine leukaemia virus enzyme, has raised efficiency in plant systems, although prime editing in cereals still trails base editing for comparable targets [15]. For a breeding programme, this efficiency gap sets a practical boundary on which natural alleles can be reproduced by design rather than sourced by crossing. The trait category does not determine platform choice. Cas9 knockout of the wheat susceptibility gene TaeIF4E conferred resistance to wheat yellow mosaic virus without a yield penalty using the same nuclease and repair pathway applied to developmental and quality targets [16]. The full range of trait applications is examined in Section 3.

### 2.2. Delivery and Regeneration as the Rate‐Limiting Step

Every editor above must be delivered into a cell that can regenerate into a fertile plant, and in cereals, this step rather than the editing chemistry sets the pace. The constraint is genotype rather than species. Barley transformation has depended almost entirely on the cultivar Golden Promise, with only a small number of additional genotypes transformable and at very low efficiency [17]. Wheat has depended comparably on Fielder. This dependence is the practical reason why many published cereal edits exist only in reference backgrounds, and it limits what those studies demonstrate about breeding application.

Four classes of solutions have been proposed, and they differ in how far each has been shown to scale. The first is coexpression of morphogenic regulators, which reprogrammes somatic cells toward an embryogenic state rather than simply accelerating regeneration in genotypes that were already responsive [18]. Controlled expression of the *GRF4-GIF1* fusion raised transformation efficiency to 77.5% in Fielder, 60% in Cadenza and 40% in Borlaug 100 and produced transformants in Skyfall and Chinese Spring, which had previously been recalcitrant or very inefficient [19]. Coexpression of *HvWUS* and *HvBBM2* extended the same principle to barley varieties beyond Golden Promise [17]. Two qualifications follow from these data. Efficiency still falls roughly twofold from the reference cultivar to an elite line, so the genotype gradient is narrowed rather than removed. Constitutive expression of morphogenic regulators also carries pleiotropic costs, including reduced fertility and spikelet number, which is why promoter choice and excision strategies determine whether the approach is usable in material intended for release [19].

The second class is delivery of preassembled ribonucleoprotein, which avoids transgene integration and therefore removes the segregation step needed to obtain a transgene‐free line. The third is transient delivery with temporary selection rather than stable integration. An accelerated microparticle bombardment pipeline for sorghum immature embryos, combining a maize‐optimised Cas9 vector with transient antibiotic selection, produced homozygous mutants in the T0 generation without a segregation step [20]. Both routes have so far been reported at a proof‐of‐concept scale in a small number of genotypes, and neither has been documented as a routine production pipeline across a breeding programme. The fourth class bypasses transformation of the target genotype altogether by delivering editing reagents through a haploid inducer, which is examined in Section 4.

Delivery format also fixes the regulatory starting position. Plasmid‐based delivery leaves an integrated transgene that must be segregated away, whereas ribonucleoprotein and transient formats do not, and this difference determines which regulatory pathway an edited line enters in each jurisdiction, as discussed in Section 6. Conventional tissue culture routes remain slow regardless of format, with months typically elapsing before an edited plantlet is recovered [21]. Table 2 records the genotype used and the level of deployment reached for representative platform applications, since these two columns, rather than the nuclease identity, determine what a study demonstrates for breeding.

**Table 2:** Representative CRISPR‐Cas editing platforms deployed in major cereal crops, showing the nuclease or editor type, target locus, trait category, the genotype in which the work was performed and the current level of deployment reached.

Platform	Crop	Target locus	Trait category	Genotype used	Deployment level	Reference
Cas9 (standard)	Wheat	*TaeIF4E*	Disease resistance	Transformable research cultivar	Confined field trial	Kan et al. [16]
Chimeric PAM‐relaxed Cas9 with adenine base editor	Rice	*OsACC*	Herbicide resistance	Transformable reference line	Laboratory proof‐of‐concept	Liang et al. [13]
Cas12a orthologs (Mb3Cas12a)	Rice, maize and tomato	Multiple loci, single and multiplex	Methodological platform	Reference lines	Laboratory proof‐of‐concept	Liu et al. [12]
Cas9, microparticle bombardment with transient selection	Sorghum	PDS	Methodological platform	Transformable inbred	Laboratory proof‐of‐concept and T0 homozygous	Zhang et al. [20]
Prime editing	Rice and wheat	Single‐nucleotide and small indel targets	Precision allele reproduction	Reference lines	Laboratory proof‐of‐concept	Gallo et al. [15]
MAD7 (ErCas12a), royalty‐free nuclease	Rice and wheat	Multiple loci	Methodological platform	Reference lines	Laboratory proof‐of‐concept, up to 65.6% editing efficiency	Lin et al. [22]
Agrobacterium delivery with controlled *GRF4-GIF1* expression	Wheat	Transformation platform	Genotype‐independent regeneration	Fielder, Cadenza, Borlaug 100, Skyfall and Chinese Spring	Platform validated across elite cultivars	Hayta et al. [19]
Agrobacterium delivery with *HvWUS* and *HvBBM2* coexpression	Barley	Transformation platform	Genotype‐independent regeneration	Multiple varieties beyond Golden Promise	Platform validated across multiple varieties	He et al. [17]

### 2.3. Freedom to Operate Across Editors and Regeneration Technologies

Access to the platforms above is governed by three distinct categories of rights, and conflating them obscures where a breeding programme is blocked. Foundational CRISPR‐Cas9 patents are held predominantly by a small number of institutions, and freedom to operate analysis has become a routine step before a nuclease platform can be deployed commercially [23]. A 2017 cross‐licensing agreement between the Broad Institute and Corteva Agriscience made foundational Cas9 patents freely available for academic research and available under a nonexclusive commercial licence for agricultural use. This arrangement does not extend automatically to downstream innovations built on that platform, and royalty obligations still apply to many commercial applications [23]. Ownership of the editing system is a separate question from access to the transformation and regeneration technology needed to use it, and the second is frequently the binding constraint in cereals. The *BBM* and *WUS2* system and the *GRF4-GIF1* chimaera are both covered by patent filings, and although research use is generally free, commercial use of *GRF4-GIF1* requires a paid nonexclusive licence [24]. Proprietary transformation systems impose a further access barrier that is procedural rather than financial, since they require specified vectors and dedicated training, which restricts uptake independently of licence cost [19]. A program may therefore hold rights to a nuclease and still be unable to deliver it into its own elite germplasm.

A third category covers the edited allele itself. Patents can be asserted over a trait or a sequence variant independently of the technology used to create it, so a program that clears both nuclease and regeneration rights may still encounter a claim on the resulting plant, and the interaction between these claims and plant variety rights differs between jurisdictions [25]. This is the category most likely to affect cereal programs that reproduce known favourable alleles by editing rather than introgression.

The practical burden of this licensing landscape falls unevenly. Research institutions and seed companies in lower‐income regions can face commercial licence costs reaching into the millions of dollars, which constrains which research groups can move a CRISPR‐edited cereal line toward commercial release even when the underlying science is complete [9]. Open‐access nuclease alternatives have emerged partly in response. MAD7, a Cas12a‐family nuclease released as royalty‐free for both academic and commercial use, achieved editing efficiencies of up to 65.6% in regenerated rice and wheat mutants and recognises a thymine‐rich protospacer‐adjacent motif distinct from that of canonical Cas12a orthologs [22]. ErCas12a, the nuclease underlying MAD7, shares only around 31% sequence homology with AsCas12a. This divergence means its intellectual property may fall outside the patent claims covering canonical Cas12a nucleases, though its editing efficiency remains strongly dependent on target sequence and cultivation temperature [26]. A royalty‐free nuclease therefore resolves only the first of the three categories above. It leaves the regeneration licence and any claim on the resulting allele untouched, which is why freedom to operate must be assessed across the whole pipeline rather than at the nuclease alone.

Platform choice, delivery route and licensing position therefore jointly determine how many genetic backgrounds an edit can reach and whether the resulting line can be commercialised. None of these constraints is resolved by a more precise nuclease. Section 3 turns to the trait applications built on this toolkit, where a further constraint appears, namely, that most cereal traits require more than one edited locus.

## 3. Multiplexed and Polygenic Trait Editing for Complex Agronomic Traits

### 3.1. Why Single‐Gene Editing Is Usually Insufficient

Most agronomically important traits in cereals are not controlled by single genes of large effect but by combinations of loci with individually modest contributions interacting within regulatory networks rather than acting as independent switches [5]. Editing one gene precisely therefore often produces a trait shift too small to be useful. Two distinct problems drive the need for multiplex approaches, and they call for different targeting logic. Functional redundancy between paralogs or homoeologs masks the phenotype of any single knockout, which requires editing all members of a family. Convergence of unrelated genes on a shared physiological output requires editing across pathways instead [4]. Comparative testing of multiplex vector architectures in wheat, including tandem repeat, tRNA‐processing and ribozyme‐processing systems for expressing multiple guide RNAs from a single construct, established that vector design itself materially affects multiplex editing efficiency in a genome as large and hexaploid as bread wheat, and this finding underlies most of the multiplex applications discussed below [27].

### 3.2. Experimentally Validated Multilocus Edits

The studies below are ordered by the level of validation reached rather than by trait category because that distinction determines what each demonstrates for breeding.

The maize BREEDIT pipeline illustrates multiplex editing applied directly to yield‐related and drought‐related quantitative traits. Using four 12‐plex constructs to target 48 growth‐related genes, the platform generated over 1000 independently edited lines and combined this multiplex editing step with conventional crossing schemes to recover fixed mutations and stack favourable alleles, with some resulting lines showing measurable increases in leaf size and drought tolerance [28]. This example is instructive for two reasons. First, a quantitative trait improvement of practical relevance required editing dozens of genes rather than one, consistent with the polygenic architecture of yield and drought response in maize. Second, the pipeline explicitly combined multiplex editing with a subsequent crossing step to fix and stack edits, which is the kind of interaction between editing and downstream breeding operations that a toolkit‐only account of CRISPR‐Cas does not capture. The qualification is that leaf size and drought response were assessed in a limited number of lines, so the platform demonstrates throughput more convincingly than it demonstrates a deployable trait gain.

A five‐gene edit in japonica rice provides the clearest example of multiplex editing carried through to a measured field outcome. A single tRNA‐processing construct targeted five loci in the cultivar Xin Dao 53, namely *GS3*, *OsPIL15* and *Gn1a* for yield components, *OsBADH2* for fragrance, and *Pi21* for blast resistance. Homozygous five‐gene lines recovered in the T2 generation and evaluated in the field at T3 showed grain number per panicle increased by 14.60%–25.61%, thousand‐grain weight increased by 3.79%–9.15%, a 42–64‐fold rise in 2‐acetyl‐1‐pyrroline and enhanced blast resistance, with no significant change in other agronomic traits [29]. This study meets the evidence criteria set out in Section 1 since it reports measured performance in a named cultivar across three generations under field conditions.

Disease resistance provides a further, mechanistically distinct illustration of why multiplex rather than single‐gene strategies is often necessary, and in this case, the necessity arises from pleiotropy rather than redundancy. Loss‐of‐function mutations in the susceptibility gene *MLO* confer durable, broad‐spectrum resistance to powdery mildew in several cereal species, but this resistance has historically come at the cost of growth penalties and yield loss. A targeted 304‐kb deletion spanning the wheat *MLO-B1* locus altered local chromatin structure in a way that ectopically activates the neighbouring gene *TaTMT3B*, a tonoplast sugar transporter, and this secondary activation alleviates the growth penalty that would otherwise accompany *MLO* loss, restoring yield in field trials whilst retaining powdery mildew resistance [30]. This case is instructive because it was not designed as a conventional multiplex edit targeting several independent genes but arose from a single large deletion with an unanticipated regulatory consequence at a linked locus, illustrating that the boundary between single‐locus and multi‐locus trait engineering is not always sharp in practice and that downstream phenotypic screening remains necessary even for edits intended to be simple. Parallel work in barley has generated novel *mlo* alleles by editing, again recovering powdery mildew resistance, and has extended the assessment to associated microbiome effects, which indicates that the consequences of susceptibility‐gene removal are not confined to the pathogen interaction itself [31].

A more deliberately multiplexed approach to disease resistance combined CRISPR‐Cas9 knockout of four negative regulators of rice immunity, *OsMads26*, *OsBsr-d1*, *OsELF3-2* and *OsERF922*, in a single construct, producing quadruple mutants with elevated resistance to rice blast and bacterial blight together with improved tolerance to drought and salt stress and with grain yield increased by between 17% and 22% relative to wild type [32]. Stacking edits across genes governing distinct stress categories can therefore produce combined biotic and abiotic resilience alongside a yield gain. This outcome remains evidence from one genetic background and one set of environments rather than a general property of the four genes targeted.

Grain quality traits governed by gene families provide a further category in which multiplex editing addresses genetic redundancy directly. Starch branching enzymes in cereals are encoded by multiple paralogous genes, and simultaneous CRISPR‐Cas9 knockout of all four starch branching enzyme genes in rice, delivered as a single construct using a tRNA‐processing system was required to achieve a substantial increase in resistant starch content, since editing any single starch branching enzyme gene alone was insufficient [33]. A parallel case concerns photosynthetic efficiency rather than grain composition. The small subunit of RuBisCO in rice is encoded by five paralogous genes, and multiplex editing of this gene family generated a series of knockout combinations to dissect which small subunit genes contribute to catalytic properties and holoenzyme stability. Single‐gene knockouts showed no measurable change in field‐grown yield or photosynthetic parameters, whilst combinatorial multiplex genotypes revealed effects that single‐gene approaches had missed [34]. Both cases show that functional redundancy within a gene family conceals the contribution of any one member and that only simultaneous editing reveals or achieves the intended shift. Table 3 summarises these against the generation reached and the environment in which performance was assessed.

**Table 3:** Representative multiplex genome editing studies in cereals for complex agronomic traits, showing the number of loci targeted, the trait category addressed, the reported outcome, the generation at which the outcome was measured and the validation environment.

Crop	Genes/loci targeted	Trait category	Reported outcome	Generation reached	Validation environment	Reference
Maize	48 growth‐related genes (four 12‐plex constructs)	Yield and drought tolerance	Over 1000 edited lines generated; some lines showed increased leaf size and drought tolerance	Fixed after crossing	Controlled environment	Lorenzo et al. [28]
Rice	*GS3*, *OsPIL15*, *Gn1a*, *OsBADH2* and *Pi21*	Yield, quality and blast resistance	Grain number +14.60%–25.61%, thousand‐grain weight +3.79%–9.15%, 2‐AP +42–64‐fold and enhanced blast resistance	T3 homozygous	Field	Guan et al. [29]
Rice	*OsMads26*, *OsBsr-d1*, *OsELF3-2* and *OsERF922* (quadruple knockout)	Combined biotic and abiotic resistance plus yield	Elevated blast and bacterial blight resistance, improved drought and salt tolerance and a 17%–22% yield increase	Homozygous quadruple mutants	Single environment	Luo et al. [32]
Rice	Four starch branching enzyme genes	Grain quality (resistant starch)	Substantial increase in resistant starch content, not achievable by single‐gene edits	Homozygous lines	Controlled environment	Biswas et al. [33]
Rice	Five RuBisCO small subunit paralogs	Photosynthetic efficiency	Combinatorial knockouts revealed catalytic and stability effects masked in single‐gene knockouts	Homozygous combinations	Field and controlled environment	Zhou et al. [34]
Wheat	Multiple loci across A, B and D subgenomes (vector comparison)	Methodological (multiplex vector efficiency)	Relative efficiency established for tandem repeat, tRNA‐processing and ribozyme‐processing systems	T0–T1	Controlled environment	Li et al. [27]
Wheat	*MLO-B1* deletion with linked ectopic activation of *TaTMT3B*	Disease resistance without growth penalty	Powdery mildew resistance retained without yield loss in field trials	Advanced homozygous lines	Field	Li et al. [30]
Barley	*Mlo*	Powdery mildew resistance	Novel *mlo* alleles recovered, with associated microbiome effects assessed	Homozygous lines	Controlled environment	Eskildsen et al. [31]

### 3.3. Biological Limits on the Predictability of Multilocus Edits

Increased molecular precision does not confer biological predictability. Epistasis, pleiotropy, genetic background effects and genotype‐by‐environment interaction all shape the expressivity of an engineered allele, and their influence grows as the number of edited loci increases [35]. The wheat *MLO-B1* case is a pleiotropy rather than a redundancy result, and an edit designed without its linked regulatory effect would have reproduced the historical growth penalty [30].

Homoeolog dosage adds a further layer in polyploid cereals. In hexaploid wheat, most genes are present as three homoeologous copies, so editing one copy frequently produces no measurable phenotype, whereas editing all three can overshoot into developmental imbalance. The relationship between edited genotype and phenotype is therefore not monotonic, and target selection in wheat must specify how many copies to edit rather than which gene to edit alone. Genetic background determines whether an effect transfers. Every outcome in Table 3 was obtained in one recipient genotype, and none has been reported across a panel of elite backgrounds. The environment determines whether it persists. Stress‐tolerance phenotypes in particular are frequently larger under controlled conditions than in the field, so a controlled‐environment result should be treated as an upper bound rather than an estimate of field performance [35].

Nitrogen‐use efficiency illustrates a related but distinct challenge, namely, that a mechanistically well‐characterised candidate gene is not automatically an editing target ready for deployment. Detailed genetic and biochemical work in rice identified a monoubiquitination pathway, centred on an F‐box protein that stabilises a negative regulator of auxin biosynthesis in roots, as a control point for root plasticity and nitrogen‐use efficiency under variable nitrogen supply [36]. Even so, translating a mechanistically validated regulatory node identified in one genetic background into a stable, field‐deployed multiplex edit in an elite cultivar remains a separate undertaking, requiring confirmation that the same regulatory logic holds in the target germplasm and environment, a distinction addressed further in Section 6.

A directly comparable illustration of this gap comes from CEP‐CEPR1 signalling, a peptide‐receptor pathway that links root nitrogen demand to root system architecture. CRISPR‐Cas9 knockout of *CEPR1* in barley produced plants with steeper seminal roots and a narrower mature root system, confirming that this pathway governs root architecture in a cereal rather than only in the dicot model systems where it was first characterised. The same knockout also produced a severe fecundity defect, indicating that disrupting this regulatory node carries a direct yield cost alongside the intended architectural change [37]. Its agronomic consequences therefore require characterisation as thorough as the mechanism itself.

Drought and other abiotic stress tolerance traits present a particularly clear case for multiplex rather than single‐gene strategies since the underlying regulatory architecture involves extensive integration across genomic, transcriptomic, proteomic, metabolomic and epigenomic layers rather than a single dominant pathway. A recent synthesis of multiomic evidence for drought tolerance in wheat, rice, maize and legumes identifies root architecture, osmotic adjustment and antioxidant capacity as convergent targets of this regulation but cautions that many candidate nodes identified through omics profiling remain correlative rather than causally validated [38]. The antioxidant example is worth stating precisely because enzymatic antioxidant activity is widely measured as a convenient index of abiotic stress tolerance despite weak evidence that it predicts tolerance reliably, which makes it a poor basis for selecting editing targets [39]. An omics‐derived candidate list is a hypothesis‐generating resource rather than a validated target set. Treating correlative associations as confirmed causal drivers commits multiplex editing effort to loci that may not control the trait under selection. A useful contrast is provided by traits where the candidate‐to‐target gap has already been closed by direct CRISPR validation. CRISPR‐Cas9 knockout of *OsEPFL9* in rice and of *SbEPFL9* in sorghum reduced stomatal density and leaf conductance without a yield penalty under drought, giving validated, field‐relevant water‐use‐efficiency edits in two cereal species [40]. The omics‐to‐target gap is therefore not a universal property of abiotic stress traits but varies with how far a given candidate has progressed toward direct genetic validation.

Cold tolerance in barley illustrates the intermediate position that many traits occupy. Adaptation to low temperature involves coordinated remodelling of membrane lipid composition in barley leaves, a response characterised at the level of lipid classes and their dynamics rather than reduced to a defined set of causal loci [41]. A mechanism described at this resolution identifies where to look for editing targets but does not yet supply them, and this intermediate state is more common across cereal abiotic stress traits than either fully validated or purely correlative evidence.

### 3.4. Editing Efficiency, Mosaicism and Stable Inheritance

Multiplex editing efficiency does not scale with the number of guide RNAs deployed, and the magnitude of the shortfall is now quantifiable. In the five‐gene rice construct described above, individual editing efficiencies ranged from 78% at *Pi21* down to 13% at *OsPIL15*, yet simultaneous editing of all five genes occurred in only 9.38% of T0 plants [29]. The recovery rate for a complete multiplex genotype is therefore set by the least efficient guide rather than by the average, which has a direct practical consequence. Population sizes at T0 must be planned against the weakest target in the construct, and guide performance should be tested individually before a multiguide vector is assembled.

A parallel synthesis of cereal abiotic stress engineering frames CRISPR‐Cas editing, marker‐assisted selection, genomic selection and multiomic technologies not as competing approaches but as components requiring coordinated deployment within a single breeding pipeline and identifies off‐target effects, delivery constraints and chimeric mosaicism, in which only a subset of cells in an edited plant carry the intended modification, as constraints that become more consequential as the number of simultaneously targeted loci increases [42]. Mosaicism is a particular concern for multiplex editing of polygenic traits since a chimeric T0 plant may carry different subsets of the intended edits in different cell lineages, and only a fraction of these lineages will contribute to gametes carrying the full complement of desired mutations. A T0 screen therefore establishes which edits are present in sampled tissue, not which edits will be transmitted, and the two can differ. Stable inheritance must be confirmed independently in at least the T1 and T2 generations, which is the standard the five‐gene rice study met by recovering homozygous lines at T2 and phenotyping them at T3 [29]. This genotyping burden connects directly to the marker‐assisted methods examined in Section 5. Figure 2 depicts this general multiplex editing strategy schematically, showing the two outcome patterns documented above, redundancy resolution and trait stacking, alongside the chimeric mosaicism problem that constrains both.

**Figure 2:**
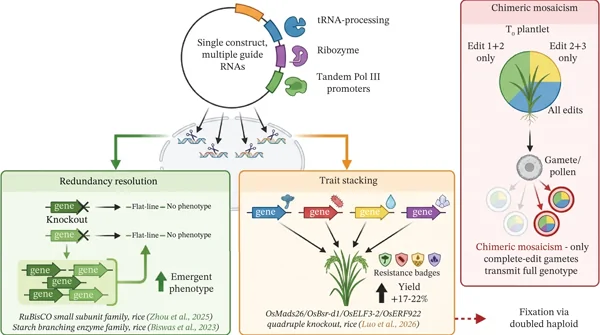
Schematic of a multiplex editing strategy applied to a polygenic agronomic trait, showing a single construct expressing multiple guide RNAs [27] directed at paralogous or functionally convergent genes. The redundancy branch is drawn from combinatorial knockouts of the rice RuBisCO small subunit family, where single‐gene edits produced no measurable change [34]. The stacking branch is drawn from the five‐gene rice construct, annotated with the fall from 78% single‐target efficiency to 9.38% five‐target efficiency [29]. The mosaicism node marks where T0 genotype and transmitted genotype diverge.

Multiplex editing has therefore become the dominant strategy for complex cereal traits, but its productive use rests on three conditions that the editing chemistry does not supply. Candidate targets must have causal rather than correlative support. Per‐target efficiency and mosaicism must be managed as multiplex scale increases. The resulting multilocus genotype must have a defined route to fixation because independently segregating edits will otherwise be lost in subsequent generations. Section 4 examines whether doubled haploid production meets the third condition in practice, and Section 5 examines the selection methods that address the second.

## 4. Coupling Genome Editing With Doubled Haploid Systems

### 4.1. Why the Fixation Problem Scales With Edit Number

Doubled haploid production offers a direct route to the fixation problem raised at the end of Section 3 since it can convert an edited gamete into a fully homozygous, nonsegregating line in a single generation rather than through the roughly six to 10 generations of selfing typically required to approach full homozygosity by conventional inbreeding. The size of the problem it addresses grows sharply with the number of edited loci, and this is the strongest argument for the combination. A primary transformant carrying independent heterozygous mutations at six unlinked loci will yield an all‐homozygous plant in one quarter to the sixth power of selfed progeny, so recovering at least one such plant with 90% probability requires screening on the order of 9400 plants [43]. Population sizes of that order are not compatible with routine glasshouse capacity, which is why fixation rather than editing becomes the limiting operation once multiplex constructs are used.

The same study demonstrated the alternative directly. Combining three multiplex constructs targeting 36 leaf growth genes with in vivo haploid induction and embryo rescue doubling produced an array of homozygous lines carrying different edit combinations within three generations, including a septuple mutant, with several genotypes showing a reproducible 10% increase in leaf size [43]. The doubled haploid step here performed a sampling function as much as a fixation function, since each recovered line represents one gamete drawn from the segregating population of edit combinations. The two established routes to haploid production, androgenesis‐based methods using anther or microspore culture and chromosome‐elimination methods based on wide crosses or haploid‐inducer lines, differ in how readily each can be paired with an editing step. Their efficiency in wheat specifically remains dependent on donor genotype and induction method rather than being uniformly applicable across germplasm [7].

### 4.2. Three Strategies for Combining Editing With Haploid Induction

Three distinct strategies have been used to combine genome editing with doubled haploid production, and they differ in where the editing step occurs relative to haploid induction. The first strategy edits a diploid parental line before crossing it into a doubled haploid pipeline. In this route, haploid induction and genome doubling serve only to fix an edit that already exists in the parent. A recent example in wheat combined this approach with RNA interference silencing of gliadin genes, generating doubled haploid lines from crosses between independently transformed RNAi and CRISPR‐edited parental lines. Multiple independently segregating modifications, silencing and editing together, were fixed within a single generation rather than through sequential backcrossing, and several resulting lines showed more than a 70% reduction in immunogenic gluten epitopes [44]. Editing frequency in the doubled haploid progeny depended on both guide RNA efficiency and parental genetic background. The doubled haploid step therefore does not simply fix whatever edit is present but inherits the genotype‐dependent editing efficiency of the parents.

The second strategy, haploid‐inducer‐mediated genome editing, delivers CRISPR reagents directly during the haploid induction process itself, either by transforming the haploid‐inducer line so that its pollen carries the editing machinery or by engineering the inducer to coexpress developmental regulators that trigger both haploid induction and genome doubling in a single step. In maize, an inducer‐mediated system targeting the *Waxy1* and *Shrunken2* genes achieved maternal haploid induction efficiencies of between 8.6% and 20.9% alongside targeted mutation rates of 0.4%–1.5%, combining two independent CRISPR‐Cas9 systems in a single inducer line to generate doubled haploid lines carrying edits at both loci simultaneously [45]. This approach directly addresses the transformation bottleneck discussed in Section 2 since an elite maize line that is recalcitrant to direct transformation can still be edited indirectly by using it as the maternal parent in a cross with an editing‐competent haploid inducer. The two efficiency figures quoted above are multiplicative rather than independent, and Section 4.4 works through what that implies for population size.

A more recent variant of this second strategy eliminates the in vitro tissue culture and chemical chromosome‐doubling steps that most haploid induction protocols still require. A maize system that ectopically coexpresses *BABY BOOM* and a cyclin D‐like gene within unfertilised egg cells produces maternally derived diploid embryos directly and, when combined with an editing step, generates gene‐edited, fully doubled progeny without tissue culture or colchicine treatment [46]. Since tissue culture dependency is itself a major source of genotype restriction and somaclonal variation in cereal transformation, as discussed in Section 2, removing this step addresses two bottlenecks simultaneously rather than only the fixation problem.

The third strategy, relevant chiefly to wheat, uses intergeneric pollination rather than a within‐species haploid inducer. Wheat ovules pollinated by maize pollen carrying CRISPR‐Cas9 reagents undergo chromosome elimination of the maize genome after fertilisation, leaving a haploid wheat embryo that has nonetheless been exposed to the editing machinery during this transient hybrid state. This embryo can then be rescued and doubled to generate an edited, homozygous wheat line without ever directly transforming the wheat genotype itself [47]. This route is particularly relevant for elite wheat cultivars that are otherwise difficult to transform directly since it transfers the transformation burden onto the maize pollen parent rather than the wheat recipient. Table 4 sets out the reported efficiencies for each of these strategies.

**Table 4:** Strategies for combining genome editing with doubled haploid production in cereals, showing reported haploid induction and editing efficiencies and the step at which genotype dependence enters.

Strategy	Crop	Editing step relative to induction	Reported haploid induction efficiency	Reported editing efficiency	Where genotype dependence enters	Reference
Edit diploid parent, then induce	Wheat	Before induction	Not separately reported	Dependent on guide RNA and parental background, over 70% epitope reduction in selected lines	Transformation of the parental line	Marín‐Sanz et al. [44]
Haploid‐inducer‐mediated editing	Maize	During induction	8.6%–20.9%	0.4%–1.5% targeted mutation	Inducer construction and cross‐combination	Li et al. [45]
In vivo induction with doubling in egg cells	Maize	During induction, no tissue culture	Not separately reported	Edited doubled progeny recovered without colchicine	Inducer construction	Ye et al. [46]
Intergeneric pollination	Wheat	During chromosome elimination	Dependent on donor genotype and induction method	Not separately reported	Induction efficiency in each cross	Qu et al. [47]
Multiplex editing followed by induction	Maize	Before induction	Reported per cross, embryo rescue doubling used	Homozygous multilocus combinations recovered in three generations	Transformation of the donor line	Impens et al. [43]

### 4.3. What Doubled Haploid Production Costs

The strategies above are frequently presented as though haploid induction converts an edited gamete into a fixed line as a matter of course. The doubled haploid literature does not support that framing, and four constraints apply independently of whether an editing step is involved.

Induction rates are low. The highest haploid frequencies reported for modern maize inducers across donor populations fall between 10% and 15%, so roughly 500 seeds from a typical donor ear yield around 75 haploids [48]. Maize is the most favourable case since in vivo maternal induction is routine there in a way it is not in wheat or barley. Haploid identification is a substantial operation, and it is error‐prone. Because inducers produce a small fraction of haploid kernels among hybrid kernels, identification is laborious, costly and time‐consuming, and it constitutes the second major step of the doubled haploid protocol rather than a formality [48]. The anthocyanin marker *R1-nj* embedded in most inducers is inhibited by dominant C1 anthocyanin inhibitors and confounded by naturally occurring anthocyanins in donor germplasm, which produces high misclassification rates and restricts the marker′s usefulness in flint, landrace and tropical material [48]. Misclassification matters more in an editing context than in conventional doubled haploid production because an edited gamete discarded as a false negative is not replaceable from the same cross.

Chromosome doubling is neither complete nor benign. Spontaneous haploid genome doubling occurs at rates too low to rely on in most germplasm, so protocols depend on cytotoxic antimitotic agents, and this dependence, together with genotype specificity and environmental modulation of phenotypic markers, constrains the scalability of current protocols [49]. Regenerated haploids remain sterile until doubling succeeds, so any haploid that fails to double is lost regardless of whether it carried the intended edit. The compound effect is visible in reported throughput. Averaged across 23 donor genotypes, overall doubled haploid production success was 13% [49]. A pipeline that recovers roughly one usable line in eight from an unedited cross does not become more efficient when an editing step with its own low success rate is inserted upstream.

### 4.4. Does the Combination Shorten the Pipeline?

The answer depends on which step is limiting, and it is not uniform across the three strategies.

For inducer‐mediated editing in maize, the two reported rates multiply. An induction efficiency of 8.6%–20.9% combined with a targeted mutation rate of 0.4%–1.5% means that at most about three kernels in a thousand, and under less favourable combinations fewer than one in two thousand, will be both haploid and edited [45]. Several hundred to several thousand kernels must therefore be screened per target to recover a single edited haploid before identification error and doubling failure are considered. This is workable where the alternative is impossible, which is the situation for an elite genotype that cannot be transformed at all, and the strategy should be judged against that alternative rather than against an idealised pipeline. For multiplex material, the calculation reverses. When the donor line is transformable and the construct targets six or more loci, the roughly 9400 plants required to recover an all‐homozygous segregant by selfing exceed the screening burden of induction by a wide margin [43]. Here, the combination produces a real saving, and the saving grows with the edit number. For a single edit in a transformable genotype, the combination is difficult to justify. A single generation of selfing will fix one locus in a quarter of progeny, which requires no inducer, no identification step and no doubling agent.

Across all three strategies, editing efficiency in the resulting doubled haploid progeny is not a fixed property of the CRISPR reagent alone but depends jointly on guide RNA efficiency, the genetic background of the parental or inducer line and the haploid induction rate achieved in that cross combination. Doubled haploid integration therefore relocates rather than removes genotype dependence from the transformation step addressed in Section 2 to the combination of induction efficiency and edit transmission addressed here. Where multiple independent edits are combined, the doubled haploid step also performs an implicit selection function, since only gametes carrying the desired combination of edits will give rise to doubled haploid lines with the complete intended genotype. Identifying which doubled haploid lines carry the full complement of intended edits requires the marker‐based genotyping approaches examined in Section 5. Figure 3 summarises these three integration strategies schematically, and the delivery, regulatory and field validation constraints that apply once a doubled haploid line carrying an edit has been recovered are addressed in Section 6.

**Figure 3:**
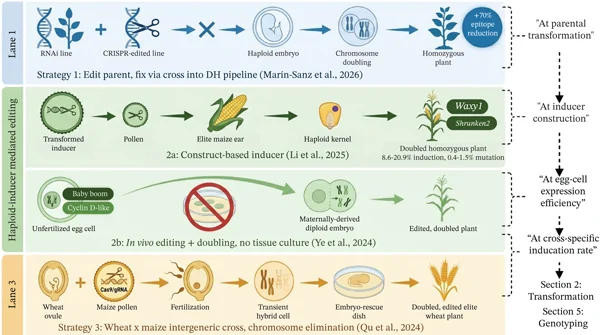
Schematic comparison of three strategies for integrating CRISPR‐Cas genome editing with doubled haploid production in cereals, annotated with the reported efficiency of each step. Strategy 1 edits a diploid parent before induction. Strategy 2 delivers reagents during induction through a transformed inducer [45] or through in vivo doubling in unfertilised egg cells [46]. Strategy 3 uses intergeneric pollination with chromosome elimination [47]. Loss points are marked at identification, doubling and edit transmission, with the entry point of genotype dependence shown for each route.

## 5. Coupling Genome Editing With Marker‐Assisted Selection and Genomic Selection

### 5.1. Where Each Method Can and Cannot Act

Once an edited allele has been fixed in a homozygous line, whether through conventional generation advance or the doubled haploid routes examined in Section 4, two further tasks remain before that line is ready for variety development. The first is confirming that the intended edit or combination of edits is present and correctly transmitted. The second is managing the genetic background surrounding the edited locus or loci since an edit introduced into a research genotype or a transformation‐amenable variety is rarely introduced directly into the elite germplasm a breeding programme wants to release. Neither task is selection in the sense that either method was designed for, and the distinction must be stated plainly because the literature frequently blurs it. Marker‐assisted selection discriminates among individuals that differ at a marked locus. A set of independently derived events carrying the same designed edit does not differ at that locus, so there is nothing for a marker to discriminate. Confirming that an edit is present and homozygous is diagnostic genotyping, and calling it marker‐assisted selection overstates what the method contributes. This review therefore treats edit verification and marker‐assisted selection as separate operations.

Genomic selection is constrained more severely still. It estimates breeding values from genome‐wide markers and requires a training population that is both genotyped and phenotyped at a scale sufficient to estimate marker effects [8]. A small number of near‐isogenic edited events supplies neither the variation nor the population size that estimation requires. Genomic selection therefore cannot act on edited material at the point of edit recovery, and any role it has must lie downstream, in the segregating populations produced once an edited line is crossed into a breeding programme, or upstream, in choosing which parents to edit. Recognising these boundaries narrows the question considerably. The remainder of this section asks what has been demonstrated within those boundaries, and Table 5 separates empirical results from simulated ones.

**Table 5:** Applications of marker‐assisted and genomic selection methods within genome editing and haploid induction pipelines, classified by what is selected on and by evidence type.

Application	What is selected or genotyped	System	Evidence type	Reported outcome	Reference
Verification of edit combinations in crossed lines	Edited progeny, diagnostic only	Wheat gliadin loci	Empirical	Deletion and insertion patterns resolved by Sanger and next‐generation sequencing, cost‐limiting at population scale	Sánchez‐León et al. [50]
Marker‐assisted backcrossing with foreground and background selection	Segregating backcross progeny	Rice blast resistance in elite japonica	Empirical	Recurrent parent genome recovery accelerated using KASP markers	Zampieri et al. [51]
Marker‐assisted selection for induction loci	Haploid‐inducer families, not edited progeny	Maize *qhir1* and *qhir8*	Empirical	TaqMan selection applied in F2 of four inducer families to raise induction rate	Khammona et al. [52]
Genomic prediction of induction ability	Haploid inducers, not edited progeny	Maize	Empirical	Multitrait prediction improved for haploid induction ability	Chen et al. [53]
Mapping to define editing targets	Mutant populations	Wheat plant height and leaf angle	Empirical	Causal intervals delimited with linked markers available for subsequent tracking	Xie et al. [54] and Yang et al. [55]
Genomic selection with promotion of alleles by editing	Simulated breeding population	Livestock, polygenic trait	Simulation	1.08–4.12‐fold greater response over 20 generations, assuming causal variants known without error	Jenko et al. [56]
Genomic selection with editing under realistic efficiency	Simulated breeding population	Livestock, monogenic trait	Simulation	At 4% editing efficiency, procedures required rose 72%, and loss in selection response rose eightfold	Bastiaansen et al. [57]

### 5.2. Demonstrated Applications of Marker‐Assisted Methods

The genotyping burden becomes concrete when multiple edited lines are crossed together to combine independently generated modifications, as in the wheat gliadin work discussed in Section 4. A related study crossed separately generated CRISPR lines deficient in gamma‐ and omega‐gliadin fractions with lines already deficient in alpha‐gliadins, using Sanger sequencing and next‐generation sequencing to characterise the resulting deletion and insertion patterns across the combined progeny, since the size and location of edits varied depending on how many guide RNA sites were engaged within a given gliadin gene cluster [50]. Once edits are combined through crossing rather than generated within a single transformation event, molecular verification of which progeny carry which combination of edits is a required step before that progeny can be advanced. This is the verification task defined above rather than selection, and its cost and computational burden, manageable for a limited number of lines, become a practical constraint across a large breeding population because they scale with population size rather than with the strength of any marker‐trait association [50].

Managing genetic background around an edited locus draws on methodology already well established for conventional trait introgression, namely, marker‐assisted backcrossing with combined foreground and background selection. In this approach, foreground markers confirm the presence of the target locus, whilst background markers distributed across the rest of the genome select, generation by generation, for individuals that retain the greatest proportion of the recurrent elite parent′s genome outside the target locus. A rice breeding programme pyramiding multiple blast‐resistance genes into an elite japonica cultivar used exactly this combined foreground and background selection strategy with KASP markers to accelerate recovery of the recurrent parent genome across successive backcross generations [51]. This is the one setting in which marker‐assisted selection operates on edited material exactly as it does on any introgressed allele because the backcross progeny segregate at every locus except the edited one. The edited allele is tracked as a foreground marker, and the remaining genome is selected as background. An edit generated in a transformation‐amenable genotype, which, as discussed in Section 2, is often not the genotype intended for release, requires precisely this operation. Where an edit is introduced directly into elite germplasm through the haploid‐inducer routes discussed in Section 4, this backcrossing burden is reduced but not eliminated since verification that the correct edit combination has been recovered still requires marker‐based genotyping of the resulting doubled haploid population.

A third application inverts the usual arrangement by applying marker‐assisted selection to the inducer rather than to the edited line. Simultaneous selection for *qhir1*, corresponding to *MTL*/*ZmPLA1*/*NLD*, and for *qhir8*, corresponding to *ZmDMP*, was carried out with TaqMan assays in the F2 generation of four inducer families derived from a tropical by temperate cross, with the aim of raising haploid induction rate and improving haploid validation [52]. This matters for the pipeline argument because Section 4 identified induction rate as a principal source of loss. Improving the inducer through marker‐assisted selection addresses that loss directly, whereas applying markers to the edited progeny does not.

Marker‐based genetic analysis also supplies editing targets rather than merely tracking them. Fine mapping of a dwarfing locus in wheat identified a mutant allele affecting plant height and delimited the interval containing it, which converts a phenotype into a defined target with linked markers [54]. Comparable fine mapping of a quantitative trait locus for erect leaf habit in the fast‐neutron‐induced wheat mutant *mths29* delimited a locus controlling leaf architecture [55]. Induced mutant populations therefore generate candidate loci that meet the causal validation standard set out in Section 3, and the markers developed during mapping remain available for tracking the corresponding edited allele afterwards. This is a more defensible route from mapping to editing than prioritising targets from correlative association data.

### 5.3. Genomic Selection Combined With Editing Rests on Simulation

No published study has applied genomic selection to genome‐edited cereal material under field conditions. This should be stated rather than implied because the proposition that editing and genomic selection reinforce one another is widely repeated and rests on a narrow and specific evidence base. That evidence base is simulation in livestock. The founding analysis, retained here because no later equivalent exists, modelled promotion of alleles by genome editing alongside genomic selection for a trait controlled by 10,000 quantitative trait nucleotides and reported that adding editing to genomic selection increased response to selection by between 1.08‐to 4.12‐fold over 20 generations relative to genomic selection alone whilst raising rates of inbreeding under some editing strategies [56]. Two features of that model limit what it establishes for cereals. It assumed that the causal variants and their effects were known without error, and it assumed editing succeeded whenever attempted.

Both assumptions have since been relaxed, with substantial consequences. Modelling the introduction of a monogenic trait under genomic selection found that editing reduced the time to allele fixation by up to fourfold when editing efficiency was complete, but that at a realistic efficiency of 4%, the number of required editing procedures rose by 72%, and the loss in selection response for the polygenic trait increased eightfold relative to the complete‐efficiency case [57]. The predicted advantage is therefore not robust to the efficiency values that Sections 2–4 report for cereals. The same work noted that promotion of alleles by genome editing is constrained in practice by limited knowledge of the causal mutations underlying quantitative variation, which is the condition cereals are in for most complex traits [57].

Genomic prediction has, however, been applied empirically at an adjacent point in the pipeline. Multitrait ridge regression prediction combined with de novo association analysis has been used to predict haploid induction ability of maize haploid inducers, which is a prediction applied to the inducer rather than to edited progeny [53]. As with the marker‐assisted example above, the demonstrated application improves the induction step rather than the editing step. The coordinated‐deployment framing noted in Section 3.4 [40] is a proposal rather than a demonstration, and it is presented here as such.

### 5.4. A Conditional Assessment

Table 5 supports three conclusions that differ from the way this combination is usually described.

Marker‐assisted methods have a demonstrated and unambiguous role, but it is narrower than the literature implies. That role is background recovery during backcrossing, target definition through mapping and improvement of the inducer. It is not selection among edited events, which is not a coherent operation. Genomic selection has no demonstrated role in cereal editing pipelines at present. Its predicted contribution comes from livestock simulations whose assumptions about causal variant knowledge and editing efficiency are not met in cereals, and the one published sensitivity analysis shows the predicted advantage degrading sharply when efficiency is realistic [57]. Treating the combination as established would misrepresent the evidence. The condition under which genomic selection would become useful is nonetheless identifiable. It requires that edited lines be crossed into breeding populations large enough to train a prediction model and that the edited locus be treated as one contributor among many rather than as the object of prediction. That is a normal genomic selection program into which an edited allele has been introduced, and it is a realistic near‐term arrangement. What it is not is a new hybrid method.

Multiplex editing, examined in detail in Section 3, places a particular strain on verification. As the number of simultaneously targeted loci increases, so does the complexity of confirming, through genotyping, which individuals in a segregating population carry the complete intended combination of edits rather than a partial subset. Where a multiplex edit targets paralogous gene family members, as in the starch branching enzyme and RuBisCO small subunit examples discussed in Section 3, marker design becomes more demanding, since closely related paralogs share sequence similarity that complicates the design of markers specific enough to distinguish edits at one family member from edits at another. This is one of several practical constraints, alongside off‐target effects and delivery limitations discussed in Section 2 and off‐target effects discussed in Subsection 6.1, that determine whether an edited line can move from laboratory generation to field‐ready cultivar, a translational question examined in Section 6.

## 6. Delivery, Regulatory and Translational Bottlenecks in Cereals

The preceding sections have each identified a point at which a technically successful edit does not automatically translate into a field‐ready cultivar. These points include delivery and transformation efficiency in Section 2, chimeric mosaicism in Section 3, genotype‐dependent induction efficiency in Section 4 and the genotyping burden of confirming multilocus edits in Section 5. This section draws these constraints together and adds two further layers, namely, off‐target characterisation and regulatory classification. It then asks how far edited cereals have travelled toward the market. The transformation constraint carries forward from Section 2 in a specific form. Efficiency achieved in a reference genotype does not transfer to the elite background a program intends to release, and the solutions compared in Section 2.2 narrow that gap without closing it. Delivery format carries forward in a different form since it determines the regulatory starting position rather than the biological one.

### 6.1. Off‐Target Characterisation

Off‐target characterisation becomes consequential once a line moves toward release because regulatory submission requires evidence that unintended modifications have been looked for. A systematic review of off‐target modifications reported across the CRISPR‐Cas plant literature found that most detected off‐target changes were small insertions, deletions or nucleotide substitutions, and that off‐target sites typically shared close sequence similarity with the intended target and were often located within homologous genes. Edited plants showed lower off‐target mutation frequencies than plants developed through conventional chemical or radiation mutagenesis [58]. That comparison sets a reasonable benchmark, since mutagenesis‐derived varieties are released without off‐target scrutiny. The comparison is nonetheless weaker than it appears because most published assessments analysed computationally predicted candidate sites rather than performing unbiased genome‐wide sequencing [58]. Predicted‐site analysis cannot detect off‐target events at sites the prediction algorithm did not nominate, so reported frequencies are lower bounds. Off‐target characterisation therefore remains a per‐line requirement for regulatory submission rather than a generalisable property of a nuclease platform, and hexaploid cereals compound the difficulty because homoeologous sequence similarity multiplies the number of plausible off‐target sites.

### 6.2. Regulatory Classification by Jurisdiction

Regulatory classification of an edited line varies substantially by jurisdiction, and this divergence is a direct determinant of which markets an edited cereal line can enter and how long that process takes. Jurisdictions fall along a spectrum between process‐based regulation, which classifies a product according to the technique used to generate it regardless of the resulting genetic change, and product‐based or outcome‐based regulation, which classifies a product according to the nature of the resulting genetic change such as whether foreign DNA remains present [59].

The global distribution is more uneven than a two‐pole description suggests. A survey of all 196 United Nations member states found 24 countries permitting these techniques for any use (Argentina being the first, in 2015), one permitting import only, 37 with legislation under discussion, three not permitting them, seven regulating them as transgenics, 114 with no legislation at all, and ten for which no data were available [60]. The largest category is therefore the absence of law rather than permission or prohibition. For a breeding programme, this is a distinct kind of risk since a jurisdiction with no legislation offers no pathway to plan against and no assurance that a pathway will not later be imposed retrospectively.

China illustrates why the wording of an approval matters. Under guidelines for the safety evaluation of gene‐edited plants introduced in 2022, the Ministry of Agriculture and Rural Affairs issued the first biosafety certificates for gene‐edited wheat and maize in 2024, followed by the first certificate for a gene‐edited rice event at the end of that year [61]. A biosafety certificate is a safety clearance of fixed duration rather than a licence to sell, and variety registration remains a separate subsequent requirement before commercial cultivation. Reporting the certificates as commercial approvals, as several secondary sources have done, overstates the position by at least one regulatory step. An edited line′s development pathway therefore cannot be planned independently of its intended markets since a line designed for one framework may require additional characterisation, different transgene‐free verification standards or a longer approval timeline in another. Table 6 records what has been reached in each jurisdiction and what remains outstanding.

**Table 6:** Regulatory status of genome‐edited crops by jurisdiction showing the primary regulatory approach, the most advanced cereal example, the deployment level it has reached and the step still outstanding before commercial deployment.

Jurisdiction	Regulatory basis	Most advanced cereal example	Deployment level reached	Outstanding step	Reference
United States	Product and outcome‐based, with exemption where the change is achievable by conventional breeding and no foreign DNA is retained	Waxy maize, multilocation field comparison against conventionally introgressed hybrids	Deregulated and commercially deployed	None	Gao et al. [62]
China	Biosafety certification under 2022 gene‐edited plant guidelines, exogenous‐sequence‐free edits prioritised	Wheat and maize certificates in 2024, first rice event at the end of 2024	Biosafety certificate granted, 5‐year validity	Variety registration before commercial cultivation	Li et al. [61]
European Union	Process‐based under the established framework, with an adapted framework for new genomic techniques under legislative negotiation	Low‐asparagine wheat trials in England, fungus‐resistant rice trial in Italy	Confined field testing only	Adoption and entry into force of an adapted framework	Lukasiewicz and Smulders [63]
Global distribution	Eight identified regulatory statuses across 196 states	Not applicable	24 states permit any use, and 114 have no legislation	Legislation in the majority of jurisdictions	Ricroch et al. [60]

### 6.3. What Has Reached the Market

The clearest measure of the translational gap is the distance between the number of laboratory‐validated cereal edits catalogued in Sections 2–5 and the number that have reached a market. In the United States, a CRISPR‐edited amylopectin‐enriched (waxy) maize developed by Corteva Agriscience was determined by USDA‐APHIS to fall outside GMO‐equivalent regulatory oversight since the edit reproduced a small deletion indistinguishable from a naturally occurring waxy allele. Field trials at 25 locations across US Corn Belt showed the CRISPR‐derived hybrids yielded on average 5.5 bushels per acre more than agronomically matched hybrids carrying the same trait introgressed by conventional backcrossing, providing a rare direct field comparison between an edited and a conventionally bred version of the same trait [62]. This remains the most complete example of a cereal edit carried from laboratory to deregulation with field data supporting the comparison, and it is notable that the advantage arose from avoiding linkage drag rather than from the edit itself.

Elsewhere the picture is thinner than the volume of publication implies. In jurisdictions where commercialisation of gene‐edited plants is legally possible, including the United States, Canada and Japan, the number of products actually on the market remains limited, and no gene‐edited plant or product had been placed on the European market as of mid‐2026 [63]. European activity remains at the field trial stage, with low‐asparagine wheat trials conducted in England as the most advanced cereal example [25].

The practical consequence of these combined constraints is that the interval between a laboratory‐validated edit and a field‐released cultivar is measured in years rather than in the single growing season required to generate the initial edit. The field trial of genome‐edited, low‐asparagine wheat discussed in Section 1 illustrates this directly. The interval between initial laboratory generation of the *TaASN2* knockout and multiyear field assessment extended well beyond the editing step itself, and regulatory consent for the field release was a separately obtained requirement rather than an automatic consequence of successful laboratory validation [10]. Figure 4 summarises these translational bottlenecks as a sequence of constraint points between laboratory demonstration and field‐ready release, and Section 7 turns to the knowledge gaps that remain across the pipeline stages examined throughout this review.

**Figure 4:**
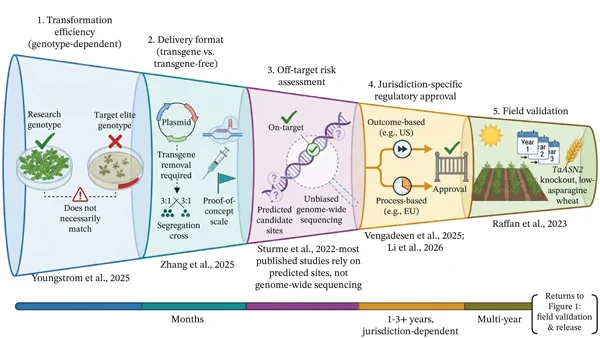
Diagram of the lab‐to‐field translational bottleneck for genome‐edited cereals showing four sequential constraint points and the deployment level at which each becomes binding. Constraint 1 is transformation efficiency in the target elite background, which does not match efficiency in the research genotype [21]. Constraint 2 is delivery format, which fixes whether a transgene segregation step is required. Constraint 3 is off‐target characterisation, limited by reliance on predicted candidate sites [58]. Constraint 4 is jurisdiction‐specific approval, branching into process‐based and outcome‐based pathways [59]. The terminal node is multiyear field validation, illustrated by the low‐asparagine wheat trial.

## 7. Knowledge Gaps and Future Directions

The evidence reviewed across this pipeline framework points to three categories of knowledge gaps that remain open despite substantial recent progress. The first concerns target validation. As discussed in Section 3, many candidate genes proposed for editing, particularly for complex abiotic stress traits, are supported by correlative omic evidence rather than direct genetic or biochemical confirmation in the target cereal. This gap between candidate identification and validated causal function has not narrowed as quickly as editing throughput itself has increased [42]. Closing this gap will require more systematic pairing of multiomic candidate discovery with functional validation in the actual crop species and genetic background intended for deployment rather than relying on generalisation from model organisms or from a single elite line. Mapping populations derived from induced mutants offers one route that already meets this standard, as Section 5 describes.

The second gap concerns scale and reproducibility across genotypes. Much of the evidence reviewed in Sections 2–4, including the haploid‐inducer‐mediated editing and doubled haploid integration strategies, has been demonstrated in a limited number of genotypes, most often reference inbred lines chosen for their transformation amenability rather than commercial elite germplasm. Whether editing efficiency, induction rates and downstream fixation success documented in these reference genotypes generalise to the broader range of elite germplasm a breeding programme works with remains an open, largely untested question for most of the studies discussed in this review. The transformation work described in Section 2.2 is an instructive partial exception since it reported efficiency separately for each cultivar and so made the genotype gradient visible rather than reporting a single figure from the most responsive background. Addressing this gap is less a matter of new tool development than of systematic testing across genotype panels, which is a less publishable but practically essential category of work.

The third gap concerns the pipeline‐integration argument this review has advanced throughout, namely, that genome editing, doubled haploid production and marker‐assisted or genomic selection are rarely reported as a single, continuous workflow within one published study. Most of the evidence assembled in Sections 3–5 draws on separate literature that each address one stage in isolation, and studies that document an edit moving through fixation, background selection and field validation within a single breeding programme remain rare. The clearest counterexamples are informative about what full integration would require. The gene editing and doubled haploid strategy in maize carried multiplex edits through to fixed, phenotyped lines, and the five‐gene rice construct carried edits through to field evaluation at the third generation, but neither documented the licensing and regulatory stages that Section 6 shows to be rate‐limiting. This is consistent with the observation made in the Introduction that recent reviews of CRISPR‐Cas technology in cereals are thorough on tools and traits but thin on pipeline integration. The gap identified there has, if anything, been reinforced rather than resolved by the evidence surveyed in the intervening sections. Table 7 makes this uneven distribution explicit across the four target species.

**Table 7:** Comparative status of each pipeline module across the four major cereals. Entries summarise the evidence cited in Sections 2–6 and indicate where a module is routine, demonstrated or not established for that species.

Pipeline module	Wheat	Rice	Maize	Barley
Editing platform maturity	Nuclease and base editing routine, prime editing less efficient, hexaploidy requires homoeolog‐aware design	Most mature, all platform classes demonstrated including PAM‐relaxed base editing [13]	Nuclease editing routine, platform work often conducted in public inbreds	Nuclease editing established, platform range narrower than rice or wheat
Multiplex and polygenic editing	Vector systems compared across subgenomes, pleiotropic outcomes at *MLO-B1*	Five‐locus construct carried to field evaluation [29]	Highest multiplex scale reported, 36–48 targets	Single and small‐scale multiplex, mlo alleles recovered
Doubled haploid fixation	Intergeneric pollination route available, induction genotype‐dependent	Rarely limiting; conventional generation advance usually preferred	Routine in vivo maternal induction, induction rate 10%–15% [48]	Androgenesis established, historically tied to responsive genotypes
Marker‐assisted and genomic selection	Marker‐assisted backcrossing standard, mapping supplies validated targets	Foreground and background selection demonstrated in elite backgrounds	Marker‐assisted and genomic prediction applied to inducers rather than edited progeny	Genomic selection well established in breeding, not yet linked to edited material
Transformation and regeneration	Genotype dependence narrowed across elite cultivars but not removed [19]	Least constrained of the four species	Morphogenic regulator systems widely used, pleiotropic costs documented	Historically confined to Golden Promise, recently extended [17]
Intellectual property and freedom to operate	Regeneration technology licensing is the binding constraint	Nuclease licensing dominant, royalty‐free alternatives available	Densest commercial patent position of the four species	Comparable to wheat, with smaller commercial sector
Regulatory pathway	Biosafety certificate issued in China, field trials in England	First gene‐edited event certified in China	Deregulated and commercially deployed in the United States	No jurisdiction‐specific cereal approval identified

Looking forward, two developments already underway are likely to shape how these gaps close, and they differ in how far each has been demonstrated in cereals. Speed breeding, which compresses generation time through controlled photoperiod and temperature regimes, is increasingly discussed as a complementary technology to genomic selection and genome editing rather than a standalone method, and its integration with editing and doubled haploid pipelines could further shorten the interval between edit generation and field‐ready line, though genotype‐specific protocol optimisation remains necessary before this integration is routine across cereal species [6]. This is a proposal supported by adjacent evidence rather than by a published cereal pipeline that has run editing, fixation and speed breeding end to end.

Computational approaches to guide design and target prioritisation sit further back still. Machine learning methods have been proposed for predicting guide RNA activity, anticipating off‐target sites and ranking candidate genes, and these functions map onto three constraints this review has identified, namely, variable per‐guide efficiency in multiplex constructs, reliance on predicted rather than unbiased off‐target detection and the weakness of correlative target selection [11]. The limitation is that predictive models are trained largely on data from model systems and reference genotypes, which is the same evidence base whose narrowness (Sections 2.2 and 3) is identified as the underlying problem. A model trained on reference‐genotype outcomes cannot be assumed to predict outcomes in elite germplasm. No published cereal breeding program has yet reported target prioritisation by these methods carried through to a validated field phenotype, so their contribution remains prospective [64].

Both directions extend rather than replace the pipeline logic developed in this review, since a faster generation cycle or a better prioritised editing target still requires the same fixation, selection and validation steps examined in Sections 4–6 before it becomes a released cultivar. The most useful near‐term contribution would be narrower and less novel than either. It would be a single published account of one edited cereal allele followed from generation through fixation, background recovery, multienvironment evaluation, licensing clearance and regulatory submission, reporting the loss at each step. Table 7 shows that every individual module has been demonstrated in at least one cereal. What has not been demonstrated is the sequence.

## 8. Conclusion

This review has treated CRISPR‐Cas genome editing in cereals as one module within a breeding pipeline rather than as a technology in its own right and has asked at each interface whether integration has been demonstrated or merely proposed. Applying that standard changes the conclusions in several places. The editing toolkit has diversified faster than the capacity to deploy it. Platform choice matters less to a breeding programme than whether the required allele is reachable, whether the elite background can be transformed and whether the regeneration technology can be licensed. Transformation efficiency in elite cultivars still falls well short of that in reference genotypes, and a royalty‐free nuclease does not resolve a licensing constraint that sits on the regeneration system or on the edited allele itself. Multiplex editing has become the dominant strategy for polygenic traits because redundancy and convergence both demand it. Its recovery rate is capped by the least efficient guide in the construct, not set by the average, and mosaicism means that a primary transformant genotype is not necessarily the transmitted genotype. Doubled haploid production repays its costs where several loci segregate independently because the alternative screening burden grows exponentially with the number of loci. For a single edit in a transformable genotype, it does not, since induction, identification and doubling each impose losses that selfing avoids. Marker‐assisted selection and genomic selection are distinct methods entering at different points. Neither acts among near‐isogenic edited events. Marker‐assisted methods act on backcross progeny, on mapped mutant populations and on haploid inducers. Genomic selection combined with editing remains supported by livestock simulation rather than cereal field data. Beyond the breeding pipeline itself, off‐target characterisation remains a per‐line requirement, and regulatory classification remains jurisdiction‐specific, with most countries still lacking any legislative pathway for edited crops. The framework proposed here is therefore not that these technologies combine naturally. It is that each interface between them imposes a measurable cost, and that the most useful next contribution would be a single documented account of one edited allele followed through every stage to release.

## Author Contributions

**Baber Ali:** conceptualisation, validation, software, data curation, formal analysis, writing – original draft, writing – review and editing. **Aqsa Hafeez:** conceptualisation, validation, software, data curation, formal analysis, writing – original draft, writing – review and editing. **Nijat Imin:** validation, data curation, writing – review and editing.

## Funding

No funding was received for this manuscript. Open access publishing facilitated by Western Sydney University, as part of the Wiley ‐ Western Sydney University agreement via the Council of Australasian University Librarians.

## Disclosure

All authors contributed significantly and have read and agreed to the published version of the manuscript. The authors reviewed and edited all outputs and take full responsibility for the content.

## Ethics Statement

The authors have nothing to report.

## Consent

The authors have nothing to report.

## Conflicts of Interest

The authors declare no conflicts of interest.

## Data Availability

Data sharing is not applicable to this article, as no datasets were generated or analysed during the current study.
